# Data on the impact of SSRIs and depression symptoms on the neural activities in obsessive–compulsive disorder at rest

**DOI:** 10.1016/j.dib.2016.05.061

**Published:** 2016-06-01

**Authors:** Yunhui Chen, Michal Juhas, Andrew J Greenshaw, Qiang Hu, Xin Meng, Hongsheng Cui, Yongzhuo Ding, Lu Kang, Yubo Zhang, Yuhua Wang, Guangcheng Cui, Ping Li

**Affiliations:** a Department of Psychiatry, Qiqihar Medical University, Qiqihar, Heilongjiang, China; b Department of Psychiatry, University of Alberta, Edmonton, Alberta, Canada; c Department of Clinical Psychology, Qiqihar Mental Health Center, Qiqihar, Heilongjiang, China; d Department of Radiology, The Third Affiliated Hospital of Qiqihar Medical University, Qiqihar, Heilongjiang, China

**Keywords:** Obsessive-compulsive disorder, Selective serotonin reuptake inhibitors (SSRIs), Depression, Regional homogeneity, Functional connectivity

## Abstract

The data provided here related to our research article (Chen et al., 2016) [1]. We provide whole-brain intrinsic functional connectivity patterns in obsessive–compulsive disorder at resting-state [1]. This article also provides supplementary information to our research article, i.e., between – group comparisons of the effect of selective serotonin reuptake inhibitors (SSRIs) and combined depression symptoms on resting-state neural activities in obsessive–compulsive disorder. The data presented here provide novel insights into the effect of SSRIs and combined depression symptoms on the neural activities at rest.

**Specifications Table**

**Table:** 

Subject area	*Psychiatry*
More specific subject area	*NeuroImage*
Type of data	*Table, figure*
How data was acquired	*3.0-Tesla GE 750 Signa-HDX scanner*
Data format	*Analyzed*
Experimental factors	*The first 10 time points were removed, slice timing, realign, normalized, smooth, detrend and filter (0.01–0.08 Hz), regional homogeneity and functional connectivity analysis.*
Experimental features	*We compared regional homogeneity and functional connectivity between 20 obsessive–compulsive disorders with SSRIs and 10 obsessive–compulsive disorders taking no medication; compared functional connectivity between 30 obsessive–compulsive disorders and 30 healthy controls controlled for the patients’ combined depression symptoms.*
Data source location	*Qiqihar, Heilongjiang Province, China*
Data accessibility	*Data is provided in this article*

**Value of the data**

• Obsessive–compulsive disorder is a disease with high heterogeneity.
• Potential confounding factors may affect the results of neural activities at rest.
• The purified sample is important in the magnetic resonance imaging research.

## 1 Data

The data is related to the abnormal resting-state functional connectivity of the left caudate nucleus in obsessive–compulsive disorder [1]. The effect of SSRIs and combined depression symptoms on the neural activities at rest was presented here. The data was acquired on a 3.0-Tesla GE 750 Signa-HDX scanner [1] and was analyzed with Data Processing Assistant for RS-fMRI (DPARSF) [2].

## 2 Experimental design, materials and methods

**Fig. 1:**
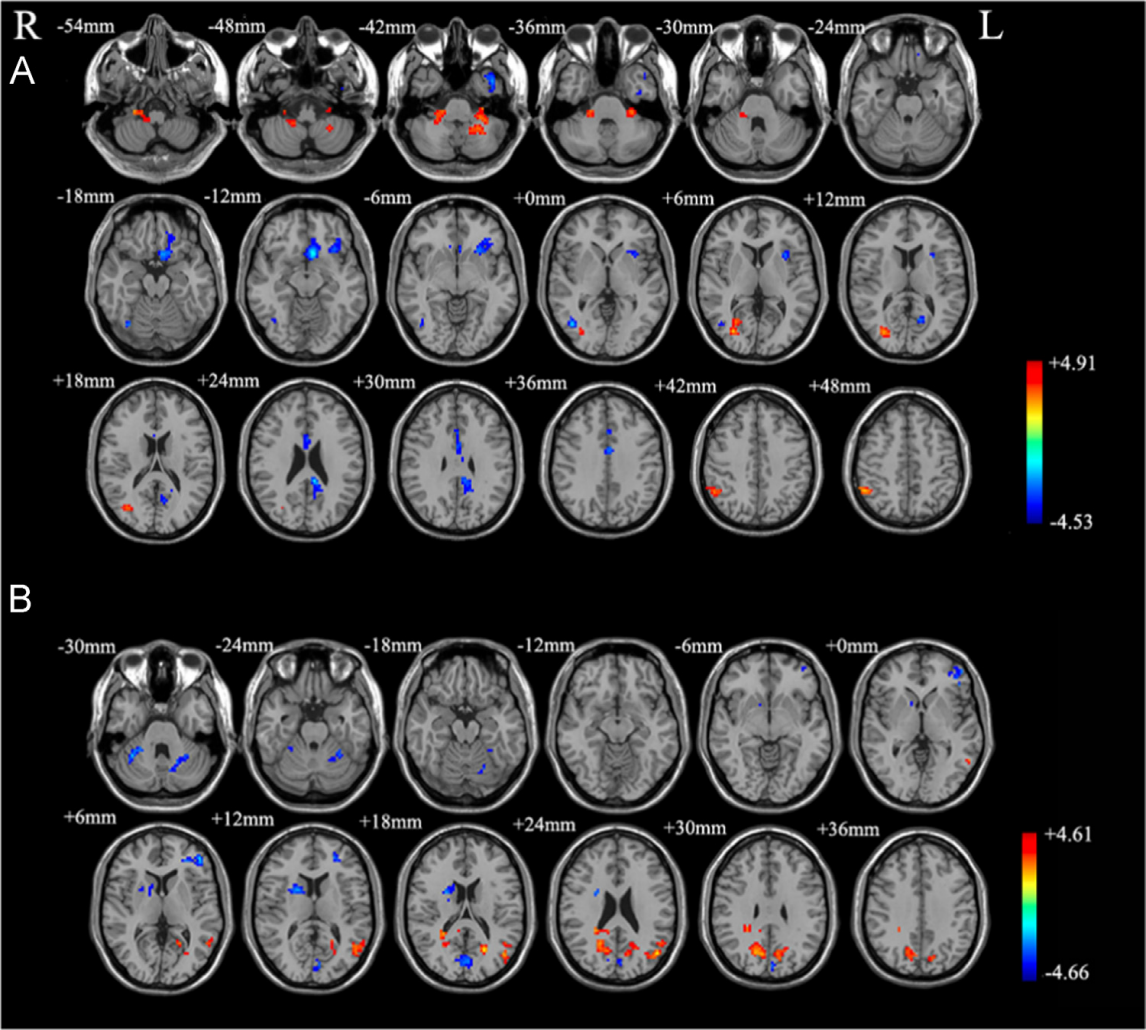
Brain regions showing abnormal ReHo (A) and functional connectivity (B) in obsessive–compulsive disorder with SSRIs. R: right; L: left; SSRIs, selective serotonin reuptake inhibitors. Red and blue denote increased and decreased ReHo. The color bar indicates *t*-value.

**Fig. 2:**
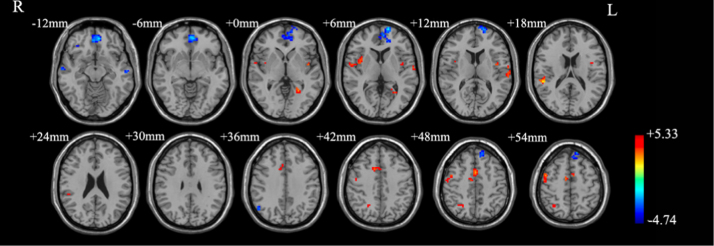
Brain regions showing different functional connectivity between obsessive–compulsive disorders and healthy controls controlled for depression. R: right; L: left. Red and blue denote increased and decreased functional connectivity. The color bar indicates *t*-value.

According to differences in functional brain connectome before and after pharmacological treatment in obsessive–compulsive disorder [3], we compared regional homogeneity (ReHo) [4] and functional connectivity between 20 obsessive–compulsive disorders with SSRIs and 10 obsessive–compulsive disorders with no medication at resting-state functional magnetic resonance imaging (RS-fMRI). Obsessive–compulsive disorders with SSRIs showed different intrinsic neural activities in local brain regions and networks, particularly in inferior frontal gyrus, temporal gyrus and inferior parietal gyrus (Table 1 and Fig. 1). According to differences in cerebral metabolism between subjects with concurrent obsessive–compulsive disorder and major depression disorder [5], we also compared functional connectivity between 30 obsessive–compulsive disorders and 30 healthy controls controlled for the patients’ combined depression symptoms and observed altered intrinsic functional connectivity in larger brain regions including the orbitofrontal cortex, dorsolateral prefrontal cortex, middle cingulate cortex, and temporal gyrus, etc. (Table 2 and Fig. 2).

## Figures and Tables

**Table 1:** Brain regions showing different ReHo and functional connectivity between obsessive–compulsive disorder with SSRIs and patients with no medication.

Hemisphere	Region	BA	Number of voxels	Coordinates of peak voxel	*t* value of peak voxel
***Increased ReHo in OCD patients with SSRIs***					
R	Inferior parietal gyrus	40	65	54, −51, 48	4.25
L	Postcentral gyrus	1	80	−48, −33, 63	3.27
L	Cerebellum		132	−27, −51, −45	3.97

***Decreased ReHo in OCD patients with SSRIs***					
R	Middle temporal gyrus	37	60	42, −69, 0	−3.94
L	Inferior temporal gyrus	20	65	−42, 6, −42	−3.33
L	Middle cingulate cortex	24	90	0, −3, 36	−3.14
L	Insula	47	117	−30, 21, −9	−3.41

***Increased functional connectivity with the left caudate nucleus in OCD patients with SSRIs***					
L	Middle temporal gyrus	39	136	−48, −69, 24	4.23
R	Cuneus		194	12, −69, 30	3.74

***Decreased functional connectivity with the left caudate nucleus in OCD patients with SSRIs***					
L	Inferior frontal gyrus	45	98	−45, 45, 6	−4.48
L	Calcarine	18	85	−6, −84, 12	−3.32
R	Precentral gyrus	6	77	33, −18, 60	−4.36
L	Supplementary motor area	6	74	−6, 12, 63	−3.87
L	Cerebellum		80	−30, −51, −27	−4.57
R	Cerebellum		56	36, −51, −30	−4.19

ReHo, regional homogeneity; OCD, obsessive compulsive disorder; BA, Brodmann area; SSRIs, selective serotonin reuptake inhibitors; R, right; L, left. (*p*<0.05, corrected with Alphasim)

**Table 2:** Brain regions showing different functional connectivity between obsessive–compulsive disorder and healthy controls controlled for depression.

Hemisphere	Region	BA	Number of voxels	Coordinates of peak voxel	*t* value of peak voxel
***Increased functional connectivity with the left caudate nucleus in OCD patients***					
R	Superior temporal gyrus	48	47	60, −3, 3	3.40
R	Superior temporal gyrus	42	27	54, −36, 18	5.33
R	Middle cingulate cortex	32	27	12, 12, 42	3.74
R	Precentral gyrus	6	32	48, 0, 51	4.07
R	Precentral gyrus	6	16	48, −9, 45	3.37
L	Supplementary motor area	6	105	−3, −3, 60	4.36

***Decreased functional connectivity with the left caudate nucleus in OCD patients***					
L	Orbitofrontal cortex	10	259	−6, 48, −9	−4.74
R	Orbitofrontal cortex	47	23	36, 33, −18	−3.89
L	Dorsolateral prefrontal cortex	9	49	−12, 45, 51	−4.42
L	Middle temporal gyrus	21	18	−60, −15, −12	−3.44
R	Middle temporal cortex	21	24	63, −12, −12	−3.65

OCD, obsessive compulsive disorder; BA, Brodmann area; R, right; L, left. (*p*<0.01, corrected with Alphasim)
