# Supplementary material for: Data on the impact of SSRIs and depression symptoms on the neural activities in obsessive–compulsive disorder at rest
Source: Data Brief. 2016 Jun 1;8:324–8. doi: 10.1016/j.dib.2016.05.061 (PMC4961786; doi:10.1016/j.dib.2016.05.061)
Supplement: Supplementary file 1 — Supplementary material [file mmc1.doc]

Conflict of Interest Form

All authors report that they have no conflict of interests.
